# Cardiac Atrial Circadian Rhythms in PERIOD2::LUCIFERASE and *per1:luc* Mice: Amplitude and Phase Responses to Glucocorticoid Signaling and Medium Treatment

**DOI:** 10.1371/journal.pone.0047692

**Published:** 2012-10-23

**Authors:** Daan R. van der Veen, Jinping Shao, Yang Xi, Lei Li, Giles E. Duffield

**Affiliations:** 1 Department of Biological Sciences, University of Notre Dame, Notre Dame, Indiana, United States of America; 2 Department of Biochemistry and Physiology, Faculty of Health and Medical Sciences, University of Surrey, Guildford, Surrey, United Kingdom; 3 Department of Physiology, Nankai University School of Medicine, Tianjin, People’s Republic of China; Pennsylvania State University, United States of America

## Abstract

Circadian rhythms in cardiac function are apparent in e.g., blood pressure, heart rate, and acute adverse cardiac events. A circadian clock in heart tissue has been identified, but entrainment pathways of this clock are still unclear. We cultured tissues of mice carrying bioluminescence reporters of the core clock genes, *period 1* or *2* (*per1^luc^* or PER2^LUC^) and compared *in vitro* responses of atrium to treatment with medium and a synthetic glucocorticoid (dexamethasone [DEX]) to that of the suprachiasmatic nucleus (SCN) and liver. We observed that PER2^LUC^, but not *per1^luc^* is rhythmic in atrial tissue, while both *per1^luc^* and PER2^LUC^ exhibit rhythmicity in other cultured tissues. In contrast to the SCN and liver, both *per1^luc^* and PER2^LUC^ bioluminescence amplitudes were increased in response to DEX treatment, and the PER2^LUC^ amplitude response was dependent on the time of treatment. Large phase-shift responses to both medium and DEX treatments were observed in the atrium, and phase responses to medium treatment were not attributed to serum content but the treatment procedure itself. The phase-response curves of atrium to both DEX and medium treatments were found to be different to the liver. Moreover, the time of day of the culturing procedure itself influenced the phase of the circadian clock in each of the cultured tissues, but the magnitude of this response was uniquely large in atrial tissue. The current data describe novel entrainment signals for the atrial circadian clock and specifically highlight entrainment by mechanical treatment, an intriguing observation considering the mechanical nature of cardiac tissue.

## Introduction

In mammals, many processes in physiology show daily variation under normal conditions. When these daily patterns persist under constant conditions, they are described as circadian (∼ 24 hours) rhythms, and are driven by an endogenous clock. Circadian rhythms are governed by internal clocks that, in the presence of timing cues, are synchronized (entrained) to the external environment [1]. The central light-entrainable clock resides in the suprachiasmatic nucleus (SCN) of the hypothalamus, and the SCN transmits timing information in a hierarchical manner to clocks found in peripheral tissues [2]. Temporal orchestration of cellular processes is seen in many peripheral tissues, such as in the liver and cardiovascular tissue, and it is thought that these tissues use a similar molecular clock mechanism as described for the SCN.

For the heart, diel (observed under light:dark conditions) and circadian rhythms are apparent in physiology and behavior. Peaks in both arterial blood pressure and heart rate in mice are observed when they are active, around Zeitgeber time 15 (ZT; ZT 0 = lights on, ZT 12 = lights off) and at ZT 12, respectively [3]. While behavioral state is an important causal factor to diurnal variation in blood pressure and heart rate, there is a significant contribution of a functional circadian clock to this rhythm [4]. A temporal profile with an early morning peak in acute adverse cardiac events and sudden cardiac death is observed in man [5], [6] and cardiac vulnerability has been found to have a circadian component that is independent of behavior [7]. Interestingly, *tau mutant* hamsters, that exhibit significantly reduced intrinsic day-length, show significant cardiac hypotrophy, reduced blood pressure and impaired myocardial contractility [8]. Also, mice that have a functional knockout of the gene encoding vasoactive intestinal polypeptide, a major SCN neuropeptide, show dampened rhythms in heart rate under entrained conditions and loss of this rhythm under constant darkness conditions [9].

A defining characteristic of circadian clocks is the expression of canonical clock genes (e.g. *clock*, *bmal1*, *per* and *cry* genes) which, through regulatory feedback loops, orchestrate each other’s activity, resulting in rhythmically active transcriptional loops [10]. Cardiac tissue has been shown to exhibit rhythmic gene expression, including the expression of canonical clock genes, *in vivo* both in the presence of an entraining light-dark (LD) cycle and under constant conditions [11]–[14]. *In vitro* cultures of cardiac tissue also show robust rhythmic expression of *per1* as reported by bioluminescence, indicating a functional circadian clock in the heart [15], [16]. Circadian variation in cardiac function persists in isolated heart tissue, highlighting the cell-autonomous activity of the cardiac clock. For example, rat hearts *in vitro* exhibit 24 hr variation in contractile responses, oxidative metabolism, oxidative stress tolerance and lipid peroxidation, and rhythmic electrochemical activity is maintained in isolated myocytes [17], [18]. Disruption of the circadian clock in the heart results in decreased diurnal variations in heart rate, sinus bradycardia, loss of diurnal variations in cardiac power and responsiveness of the heart to changes in workload, increased fatty acid oxidation, decreased cardiac efficiency, as well as other cellular functions [19].

Clocks in peripheral tissues are entrained in part, by the central light entrainable clock in the SCN, and there are factors in serum that can reset peripheral clocks such as in liver and fibroblasts cells [20]–[23]. Glucocortcoids (GCs) have been implicated as being important in synchronizing clocks in peripheral tissues *in vivo* and *in vitro* [24]. Indeed, there is evidence for direct binding of the glucocorticoid receptor (GR) to *per1* and *per2* genes, and binding of the GR to *per2* is essential for amplitude regulation by the synthetic glucocorticoid dexamethasone (DEX) in *in vitro* cultures of mesenchymal stem cells, and maybe even for the maintenance of a peripheral rhythm *per se* [13], [25]. In response to acute stress, *per1* mRNA, but not *per2* mRNA is readily upregulated in the heart, and this is subject to the presence of the GR binding sequence in the promoter region of the *per1* gene [13].

GCs are steroid hormones that are released by the adrenal gland. The release of GCs is under the control of the hypothalamic-pituitary-adrenal (HPA) axis and GCs show a daily rhythm in circulating plasma levels. Plasma levels of corticosterone, a major GC, peak at the onset of activity, both in nocturnal and diurnal subjects. As the main light-entrained circadian oscillator, the SCN drives rhythms in plasma GCs, and when the SCN is lesioned, the daily rhythm in circulating corticosterone is ablated [26]. The SCN acts on the adrenal gland, both through direct sympathetic connections to the adrenal cortex, and through modulating adrenocorticotropic hormone (ACTH) release via rhythmic release of corticotrophin-releasing hormone from the hypothalamic paraventricular nucleus (For review see [27]). The sympathetic SCN-adrenal pathway is hypothesized to control the adrenal sensitivity to circulating ACTH [28]. Besides SCN control of daily rhythms in GCs levels, there is also a role of clocks in the adrenal gland itself.

Most canonical clock genes show rhythmic expression in the adrenal gland and the adrenal clock regulates the sensitivity of the adrenal gland to ACTH, varying with time-of-day [29]. Moreover, the local adrenal clock has been reported to cause rhythmic expression of adrenal steroid production, and absence of the adrenal clock reduces daily amplitude of steroid production [30]. This reduced amplitude could be indicative of a role of GCs in synchronizing clocks in peripheral tissues [24], where desynchronized tissue cause an attenuation of overall amplitude of the clock and clock output.

Circadian rhythms are apparent in cardiac tissue at the molecular and functional level. It has been shown previously that DEX can affect clock gene expression in the heart [24], although this has been tested only at one particular time of day. Because of the reported susceptibility of clock genes to GCs, we set out to study the response of atrial tissue to dexamethasone (DEX) in a time informed manner. Using *in vitro* atrial tissue cultures of mice carrying bioluminescence reporters for *per1* and PER2 activity, we monitored the circadian clock in this tissue, and it’s response to DEX treatment. *In vitro* culture systems allow for true examination of tissue-specific circadian responses independent of extrinsic systemic factors. In these studies we specifically focused on the cardiac atria. The cardiac atria are multifaceted structures that function in the circulatory system to promote continuous venous flow; they contain pacemaker elements required for timing of heart rate; and function as an endocrine organ, producing atrial natriuretic peptide in response to stretching of the atrial vessel walls associated with elevated blood pressure or volume [31], [32]. Because DEX reportedly does not shift the rhythm of *per1* expression in the SCN (although only tested at one time of day), but has a known response in liver [24], we also cultured SCN and liver of the same individuals.

## Results

**Figure 1:**
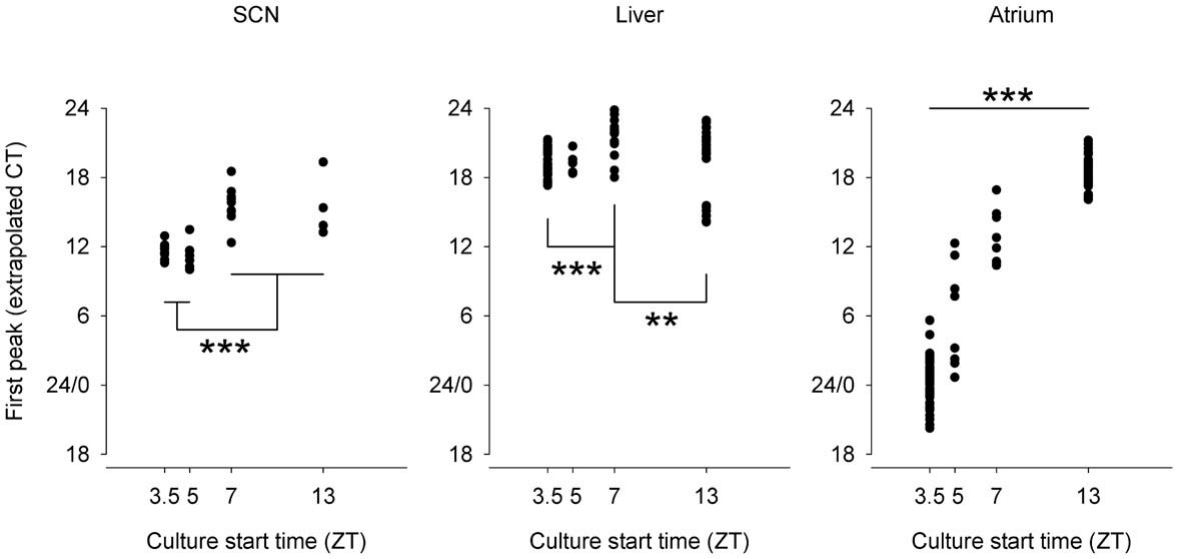
First peak in the bioluminescence rhythm in cultures of SCN, liver and atrial tissue of PER2^LUC^ mice as a function if time of culturing. Time of culturing is denoted as Zeitgeber time (ZT), where ZT0 = lights on and ZT12 = lights off, and the first peak as extrapolated CT (similar as ZT, but based on the preceding light-dark cycle). Explants were cultured at ZT3.5, ZT5, ZT7 and ZT13, and all tissues examined showed an effect of time of culturing on the subsequent *in vitro* phase, where culturing atrial tissue showed the strongest influence of time of culturing, with all times of culturing resulting in a different phase *in vitro*. **P<0.01, ***P<0.001.

The peak phase of the circadian rhythm of *in vitro* bioluminescence shows variation as a result of the time of sacrifice and subsequent culturing. Figure 1 shows that in cultures of the SCN, liver and especially atrial tissues the phase of the first PER2^LUC^ driven peak *in vitro* is different between groups with different times of killing and culturing (One way ANOVA; SCN, F_3,26_ = 18.442; liver, F_3,74_ = 5.384; atria, F_3,101_ = 499.783; P’s <0.002 for culture start time). In the case of atrial tissue, the peak phase of PER2^LUC^ bioluminescence rhythm is actually different between each of the four culture start times. In order to test for a possible ‘resetting’ of the clock, the time interval between start of culturing and the first peak was calculated, and shown to be different for different times of sacrifice for each of the tissues (Kruskal–Wallis one-way ANOVA; SCN, H_3_ = 16.002; liver, H_3_ = 56.333; atria, H_3_ = 70.306; P’s <0.001 for cultures start time). These data indicate that an *in vivo* treatment followed by *in vitro* culturing at different times of day is not a suitable approach for testing the effects of DEX on atrial or other tissues in a time-informed manner, we therefore continued with an *in vitro* treatment approach, where time of treatment is determined by the phase of bioluminescence after culturing.

**Figure 2:**
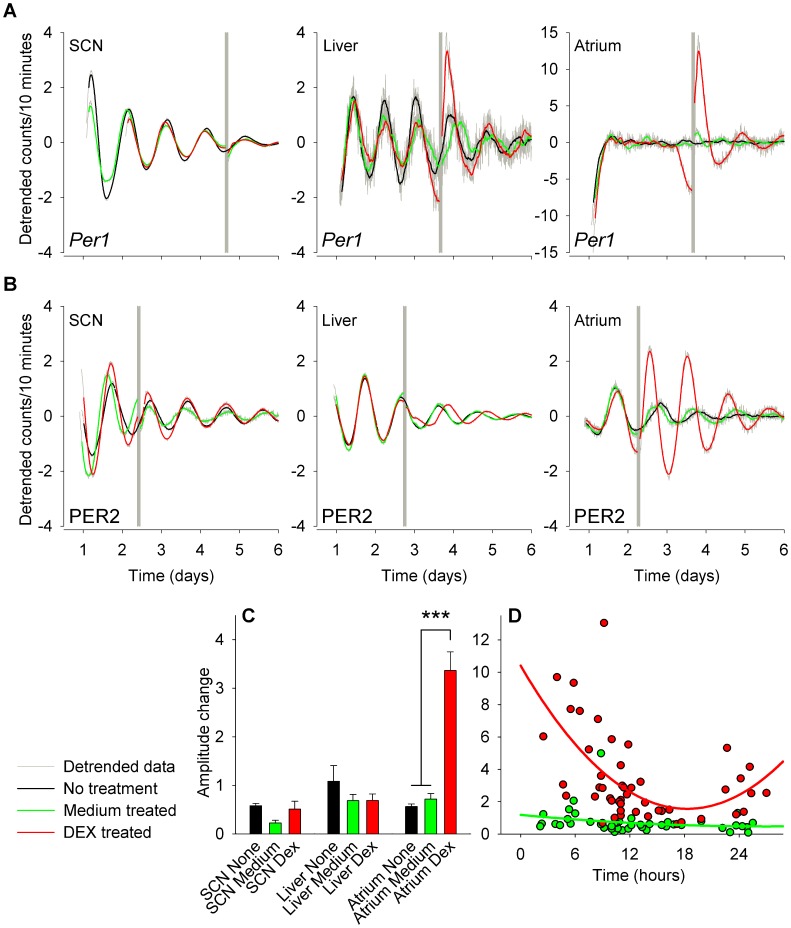
Individual baseline detrended bioluminescence recordings of *per1^luc^* and PER2^LUC^ mice in cultures of SCN, liver and atrial tissue. Data are explants derived from (A) *per1^luc^* and (B) PER2^LUC^ mice. Data are plotted as smoothed data (3 hours running average, colored lines) on top of a background of the unsmoothed data in gray tone. Grey blocks indicate time of treatment for the medium and DEX treated cultured. Amplitude responses to medium and DEX treatment for cultures of PER2^LUC^ mice are shown in panel C. The amplitude changes in cultures of atria are further plotted in panel D, showing the responses at different times of day. ***P<0.001. Note the different scale on the atrial traces of *per1* bioluminescence due to absence of pre-treatment rhythmicity.

Using this *in vitro* approach we first cultured tissues and then subjected them to control or experimental conditions. Representative baseline subtracted traces for *per1* and PER2 driven bioluminescence are shown in Figure 2A and 2B respectively. Traces are shown for SCN, liver and atrial cultures that received no treatment, or were treated with medium or DEX. As a first observation, no rhythms in *per1^luc^* bioluminescence were detectable in the atria. In contrast, PER2^LUC^ bioluminescence was rhythmic in all tissues. This observation is quantified in Table 1, which indicates the significant lack of rhythmic cultures of *per1^luc^* bioluminescence as compared to PER2^LUC^ bioluminescence (chi-square, χ_1_ = 134.111, P<0.001). The fraction of cultures that showed rhythmicity was 97% for the SCN and 82% for liver cultures and was not different between *per1* and PER2 driven bioluminescence (chi-square, χ_1_ = 1.525 and χ_1_ = 0.486 respectively, P’s >0.05).

**Table 1:** The proportion and percentage occurrence of rhythms of *per1^luc^* or PER2^LUC^ bioluminescence observed in cultures of SCN, liver and atrium.

	*per1^luc^*	PER2^LUC^	Rhythmicity		P
**SCN**	28/30	22/22		97%	NS
**Liver**	36/45	83/98		82%	NS
**Atrium**	4/45	116/118		9% vs 98%	<0.001

As a first response to treatment, we quantified the change in the amplitude of the oscillation in bioluminescence in response to no treatment, and medium or DEX treatment. DEX treatment increased the amplitude of the *per1^luc^* bioluminescence oscillation in cultures of liver, but not SCN (two way ANOVA, effect of tissue F_1,23_ = 35.208; effect of treatment F_2,23_ = 21.292; interaction effect F_2,23_ = 16.071, P’s <0.001), an amplitude increase in atrium is apparent, but could not be quantified due to absence of a rhythm before treatment (Figure S1). Although already slightly increased in response to medium treatment, DEX treatment resulted in a 4-fold increase in amplitude, which was not dependent on time of treatment (Holm-Sidak; P>0.05; data not shown). The amplitude increase for atrial tissue in response to DEX was not quantifiable due to a lack of pre-treatment rhythmicity. Because of the lack of oscillations in atrial *per1^luc^* bioluminescence, we continued to look at PER2^LUC^ bioluminescence. Figure 2C shows amplitude responses in PER2^LUC^ bioluminescence in SCN, liver and atrial cultures, where only in atrial tissue a significant increase in amplitude was observed (two way ANOVA, effect of tissue F_2,200_ = 8.235, P<0.001; effect of treatment F_2,200_ = 5.858, P<0.005; interaction effect F_4,200_ = 11.725, P<0.001). The *post hoc* contrasts indicated that the only significant change in post-treatment amplitude is observed in the atrium after DEX treatment (Holm-Sidak; P<0.001 for DEX versus no treatment and medium treated). Interestingly, while there is an overall >3-fold increase in amplitude, the response of the atrium to DEX is time dependant, in contrast to the medium treatment (Figure 2D; PROC mixed: effect of treatment, F_1,51_ = 47.78, P<0.0001; effect of time, F_21,51_ = 2.04, P<0.05; interaction effect, F_19,51_ = 1.79, P = 0.05), where DEX treatment shows a significant increase versus medium treatment when treated 2, 3, 5, 6, 9 & 10 hours after the last peak in bioluminescence. While there were already no significant increases in overall amplitude responses in liver and SCN, testing for differential treatment, time-of-day, or interaction effect for either SCN or liver tissue, revealed no significant effects (P’s >0.05), indicating that the overall absence of an amplitude response was not masking a time-of-day specific amplitude response in these tissue.

**Figure 3:**
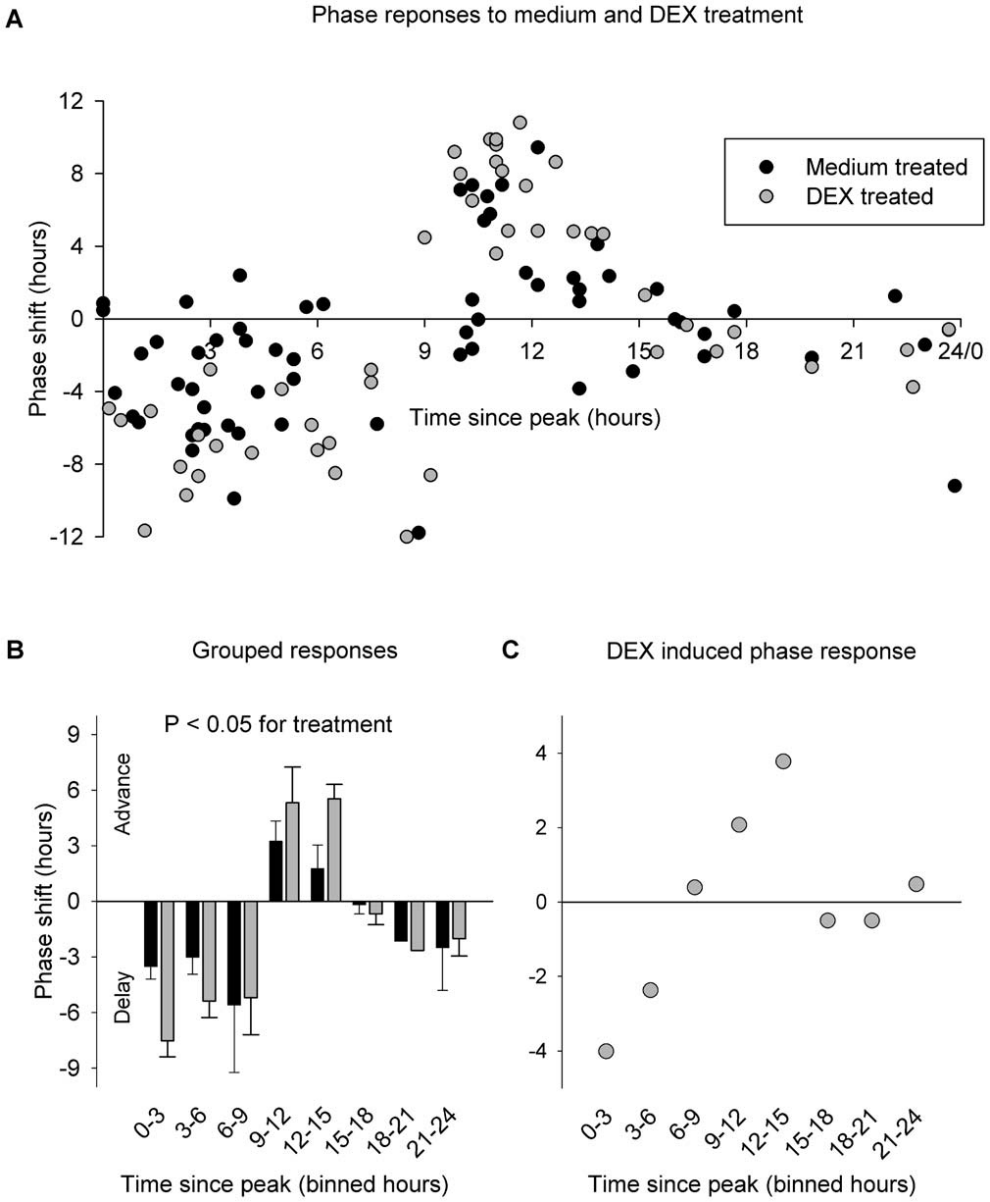
Phase shift responses to medium and DEX treatment of cultures of atrial tissue taken from PER2^LUC^ mice. (A) Data are plotted as phase shifts to treatment given at different times after the last peak in bioluminescence. For statistical analysis the data were grouped in 3 hour bins (B) and there is a significant effect of treatment, where DEX treated cultures always showed a larger phase shift than medium treated cultures. Panel C shows a DEX-only PRC, as calculated by subtracting the medium response from the DEX response as binned in panel B.

Not only amplitude, but also phase of the rhythms of tissues was affected by medium and DEX treatment *in vitro*. Figure 3A shows the phase shift response of individual atrial cultures when treated *in vitro* with medium or DEX at different time intervals after the peak in PER2^LUC^ bioluminescence. Within 0 to 9 hours following the peak phase of the rhythm, phase delays were observed in the atrium, whereas 9 to 15 hours after the peak phase, shifts in the rhythm were characterized by phase advances. Between 16 and 18 hours after the peak phase, the phase shifting responses of both medium and DEX consist of minor variations around 0 hr, which is typically referred to as the ‘dead zone’ of a phase response curve. These shifts are binned in 3 hours (Figure 3B) and the absolute magnitude of the phase shifts differs both between different times of day and between the treatment type (two-way ANOVA, effect of time F_7,96_ = 6.198, P<0.001; effect of treatment F_1,96_ = 5.327, P<0.05 for treatment; non-significant interaction, F_7,96_ = 1.730, P>0.05). The phase responses for DEX were different from those of medium, and Figure 3C shows the phase responses that are attributable to DEX through subtraction of medium induced responses. In this format, a dead zone for DEX-specific responses extends from between 16 and 23 hours after the peak phase. Interestingly, there is a limited, but significant positive linear relationship between amplitude and phase response, where a larger amplitude increase after treatment predicts a larger absolute phase shift (Linear regression R^2^ = 0.14; F_1, 35_ = 5.6409, P<0.05).

**Figure 4:**
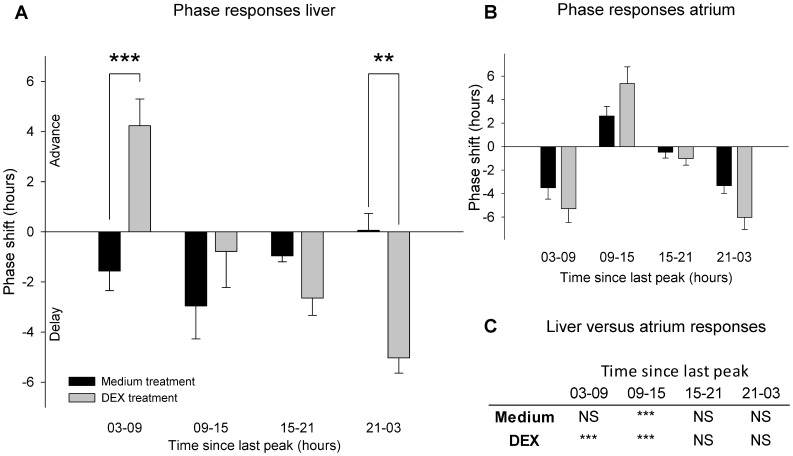
Phase shift responses to medium and DEX treatment of cultures of liver tissue derived from PER2^LUC^ mice. (A) Data are binned in 6 hour bins centered on 6, 12, 18 and 24 hours after the last peak in bioluminescence and responses differ between medium and DEX treated tissue, specifically for the phase responses after treatment centering around 6 and 24 hours after the last peak in the bioluminescence rhythm. For reasons of comparison, atrial phase responses to medium and DEX treatment are re-plotted on the same time scale as the liver in panel B. The liver and atrium show markedly different responses to treatment, and a statistical comparison between treatment and tissue is summarized in panel C. NS = not significant, **P<0.01, ***P<0.001.

As a reference, we also looked at the phase shift responses of liver tissue in response to treatment with medium and DEX at different time points centered around 6, 12, 18 and 24 hours after the last peak in bioluminescence. The phase responses are shown in Figure 4A and a significant effect of time of treatment and an interaction between time*treatment was observed (two-way ANOVA: effect of time, F_3,40_ = 7.419, P<0.001; effect of treatment, F_1,40_ = 0.194, P>0.05; interaction effect, F_3,40_ = 12.333, P<0.001). Around 6 hours after the last peak in PER2^LUC^ bioluminescence, the phase delay in response to medium treatment was significantly different from that of DEX, which caused a large phase advance (Holm-Sidak, P<0.001). When treated 24 hours after the last peak, DEX treatment now resulted in a large phase delay, which is significantly different from the very small phase advance in response to medium treatment (Holm-Sidak, P<0.005). The phase responses in the liver (Figure 4A) were different from those observed in the atrium, which is replotted on the same time-scale in Figure 4B. A significant difference in phase shifts was observed between treatment of atrium and liver with medium and DEX (PROC mixed: interaction effect of tissue, treatment and time F_6,144_ = 4.26, P<0.001).The responses to medium treatment differ between atria and liver when treated between 9–15 hours only (LSmeans, t_144_ = 3.34, P<0.005). Contrastingly, DEX treated liver and atria show tissue-specific responses when treated between 3–9 and 9–15 hours (LSmeans, t_144_ = −5.49, P<0.001 and T_144_ = 3.36, P<0.005 respectively, see Figure 4C).

Because the SCN has been reported to not shift in response to DEX treatment, we included SCN cultures as a control. We examined whether there was a phase shift in *per1^luc^* and PER2^LUC^ bioluminescence in response to medium and DEX treatment. Both treatments resulted in minimal phase shifts (medium, 0.14±0.81 hr; DEX, −0.01±0.94 hr; mean ± SEM) and our data indicate that indeed there was no effect of DEX treatment, as compared to medium treatment (two-way ANOVA: effect of time, F_3,16_ = 0.156, P>0.05; effect of treatment, F_1,16_ = 1.417, P>0.05; Figure S2).

**Figure 5:**
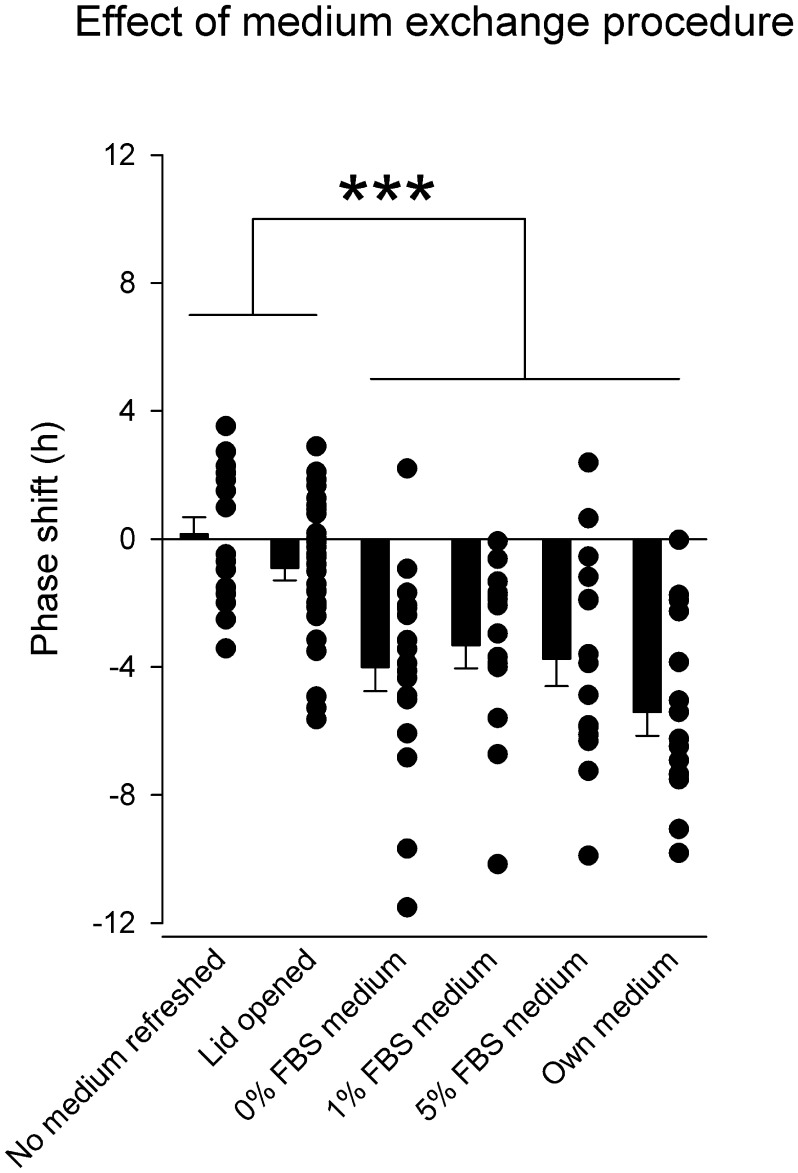
Effect of medium exchange procedure on the magnitude of phase shifts of atrial explants. Data are phase responses of cultures of atrial tissue of PER2^LUC^ mice to treatment with gas exchange only (tissue culture lid opened), medium exchange with different serum concentrations (0, 1 and 5%) or removal and reapplication of own medium. Treatment time was at 3.75±0.28 hours after the last peak of bioluminescence (mean ± SEM; range 1.0–5.0 hours). Group means ± SEM values are indicated by filled bars, and individual data points contributing to these averages are shown as circles. **P≤0.01.

The large responses to medium treatment, especially in the cultures made from atrial tissue, were striking, and we wished to investigate whether these responses were related to the mechanical movement during the media change procedure and/or serum content or change in gas pressures in the air-tissue/medium interface. We cultured atrial tissue and removed them from the incubator at 3.75±0.28 hours (mean ± SEM; range of 1.0–5.0 hours) after the last peak, a time at which we expect to see a moderate phase delay in response to the media treatment. When we did not treat the atria but simply removed the tissue from the incubator, we observed no net phase shift, 0.16±0.52 hour (mean ± SEM), in PER2^LUC^ bioluminescence (Figure 5). Similarly, removing the tissue from the incubator but opening the lid of the sealed tissue culture dish to provide for air exchange, did not result in a phase shift comparable to that of medium treatment, −0.90±0.39 hours (mean ± SEM). When we treated the tissue with fresh medium containing 0%, 1% or 5% serum (5% being the standard concentration used in all other experiments) or its own medium, we observed phase delays, and with similar magnitudes, −4.13±0.04 hours (mean ± SEM). The phase delays seen in all four medium treatment groups was significantly different from the limited phase response of untreated (but moved) or lid opened atrial cultures (one-way ANOVA, F_5,102_ = 10.836, P<0.001). Post hoc Holm-Sidak t-tests reveal that all four medium treatment groups showed significant phase delay responses to media treatment as compared to the removal from incubator only and lid opened groups (both no medium exchanged or lid opened only versus all media treatments, P≤0.001). Interestingly, no difference was observed in the magnitude of phase shifts between the four different medium groups (P>0.05), suggesting that the change in media type, namely fresh versus original media, or the presence/concentration/absence of serum in the media, is not causative for this phase shift response of the atria. Moreover, the absence of phase shifts in the lid opened group also suggests that the phase shift response is not due to partial pressure changes in O_2_, CO_2_ and H_2_O in the air surrounding the surface of the tissue and culture medium.

Because of the differences observed in the atria in the pronounced occurrence of robust rhythms in PER2 expression but absence of *per1* expression rhythms, we wished to examine this phenomenon in another *in vitro* peripheral tissue system. We used NIH3T3 fibroblast cultures to look at rhythmicity of *per1* and *per2* gene expression induced by serum treatment (Figure S3). Analysis of *per1* revealed an acute effect of the serum treatment (one factor ANOVA for time = 0–48 h, F_13,42_ = 2.6, P<0.01; time = 0–8 h, F_3,12_ = 7.1, p<0.01) with mRNA levels being induced 1h following serum stimulation (post hoc Dunnett's t-test, p<0.05) and then decreasing back to non-fluctuating levels by 4 h (P<0.05). Analysis of *per2* revealed an effect of the serum treatment upon mRNA levels (one factor ANOVA, F_13,68_ = 8.4, P<0.001). The levels of *per2* were induced transiently peaking 1 h following the start of the serum treatment (post-hoc Dunnett's t-test, p<0.05) and decreased to baseline levels by 8 h post serum (p<0.01), then rose to peak again 24 h later (12 h v. 24 h, p<0.01) and decrease to low levels again by 36 h (p<0.01) rising again at 48 h. These data reveal a significant acute induction of both *per1* and *per2* gene expression in response to serum treatment, but a significant rhythm was observed for *per2* only.

**Figure 6:**
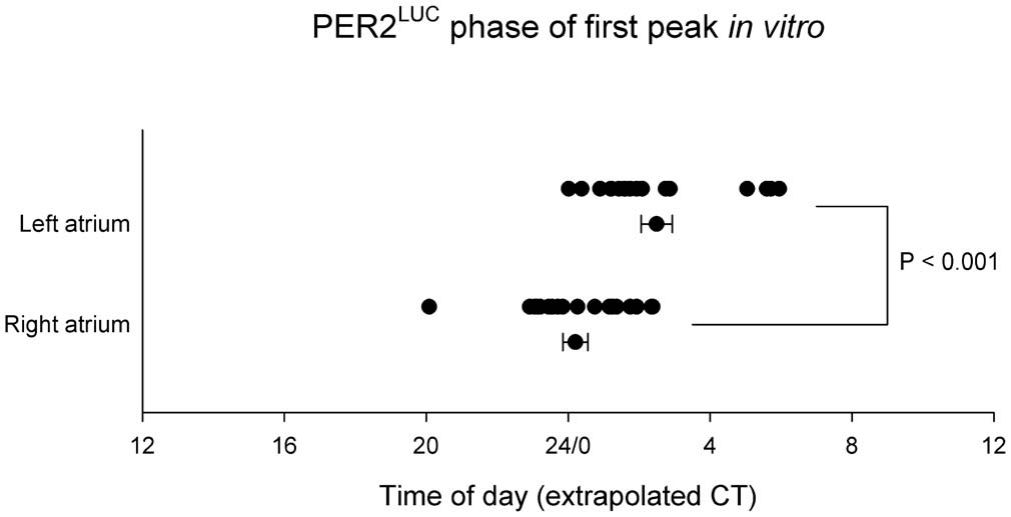
Timing of the first peak in the bioluminescence rhythm in cultures of left and right atrium of PER2^LUC^ mice. The phases for individual cultures and mean ± SEM are shown. The phase is plotted as Zeitgeber time (ZT), based on the preceding light-dark cycle, where ZT0 = lights on and ZT12 = lights off.

We made one other observation on the peak phasing of the PER2^LUC^ bioluminescence rhythm in cultures made from atrial tissue. As shown in Figure 6, the mean phase of the PER2^LUC^ bioluminescence in the right atrium was more than 2 hours (and 7 minutes) later than the left atrium (t-test, t_35_ = 4.1, P<0.001).

## Discussion

In our initial *in vivo* approach, we discovered that time of culturing influences post-culturing peak time of the PER2^LUC^ bioluminescence rhythm. This effect was strongest in atrial tissue, but was also evident in SCN and liver tissue. It is important to note that in the atrial tissue, phases of the PER2^LUC^ bioluminescence rhythm are shifted within 24-hour range when they were cultured at times with a 12 hour range. This indicates that the clock in the atrium is not ‘simply’ *reset* by culturing (which would result in a 12 hour range, instead of a 24-hour range of shifts), but that the phases found are the results of *shifts* in the atrial clock. That the clock is not reset is further corroborated by the fact that the interval between culturing and the first peak is dissimilar between the different times of culturing. Since PER2 represents a state variable of the clock, one might predict other clock components would also be affected similarly, if not immediately, certainly later [33].

Also in atrial cultures from *per1:luciferase* (*per1:luc*) rats, an effect of time of culturing has been observed when prepared at two different time points six hours apart [16]. The peak phase of cultured rat *per1:luc* SCN tissues also shows an effect of time of culturing, with a range of 6 hours [34], which is similar to the range we find in the SCN in the current study in mouse PER2^LUC^ bioluminescence. In the same study also rat pineal and pituitary (but not cornea) tissue peak phase *in vitro* exhibits modulation by time of culturing. The susceptibility of tissue to shifting effects due to culturing time appears to correlate with the amplitude of oscillation, which may be indicative of oscillator stability [34]. The large phase shifts that we see in the atrium may indicate that the clock in the atrium is less robust than those in the liver or SCN, but may also indicate a specific atrial sensitivity to mechanical stress (see later).

We continued our study with an *in vitro* approach, and cultured all tissues at the same time of the circadian day. An initial observation we made was that when we culture the mouse atrium, *per1^luc^* bioluminescence showed no significant circadian rhythm. A 30 minute DEX treatment resulted in a large peak in bioluminescence, and in many cases one or more ‘cycles’ of *per1^luc^* bioluminescence followed the DEX treatment of these atria. In contrast, in rat *in vitro* cultures of atrial tissue, *per1* driven bioluminescence is reported to be rhythmic [16]. So and colleagues [25] also report DEX induced expression of *per1* and *per2* in mesenchymal stem cells *in vitro*, but the transcriptional levels of *per1* in untreated cells is markedly less rhythmic than *per2*, if rhythmic at all, which is in line with our results. The induction of an oscillation in constitutively expressed *per1* by DEX treatment, is in line with the notion that humeral signals phase-synchronize individual cells within a tissue [35], [36]. These data also suggest that some of the dampening of rhythms in *per1^luc^* expression observed in peripheral tissue explants in culture may be explained by not only a dissociation of coordinated rhythmicity of individual cells but also by the loss of rhythmicity within the individual cells. Since this loss is most apparent in tissues derived from the *per1:luc* rat and less obvious in the PER2::LUC mouse [21], [37] would be consistent with our data that also show a robust rhythm in atrial PER2^LUC^ but absence in atrial *per1^luc^*. Additionally, the *per1* rhythm observed *in vivo* in the heart [11]–[14], but which is absent in our study *in vitro*, is likely to be driven by signals derived from outside of the tissue, i.e. it is a “systematically driven gene” [38]. A limitation to this interpretation is that whilst our study focused on the atria, the *in vivo* studies examined tissues harvested from the ventricle or whole heart.

Whilst the circadian clock in immortalized fibroblasts has been shown to be synchronized by serum treatment [20], [23], [39], in the current study in mouse NIH3T3 cells we observe a discrete rhythm in *per2* gene expression but the absence of a significant rhythm in *per1*. This is consistent with other studies of synchronized NIH3T3 cells that show either an absence of significant *per1* rhythmicity ([40]–[42]; Best, J.D., Duffield, G.E., Loros, J.J. and Dunlap, J.C., unpublished data) or a low amplitude/dampening rhythm [39], [43], [44], whilst simultaneously reporting discrete rhythms in other clock genes, e.g. *per2*, *per3*, *cry1* and *bmal1*. Interestingly, this observation is consistent with the results of our PER2^LUC^ and *per1^luc^* analysis in cultured atria, and provides yet more evidence for cell and tissue-dependent organization of the clock.

We further continued to explore the PER2 expression in atrial, liver and SCN tissue in a time informed manner. While the amplitude of PER2 driven bioluminescence is not significantly altered by DEX treatment in SCN and liver tissue, atrial tissue shows an on average more than 3-fold increase in bioluminescence. The observation that the SCN is not responsive is in line with the literature (see [24]). We are unaware of any reports that report the differential amplitude response of PER2 to DEX treatment in liver and atrium that we observe. On further analysis, we found that the amplitude response was strongly dependent on time of treatment. The amplitude response curve shows a maximal amplitude response when atrial tissue is treated with DEX around the time of intrinsic peaking of bioluminescence, and a low to absent amplitude response to DEX treatment is observed around 18 hours after the last peak.

Both the *per1* and *per2* genes contain glucocorticoid receptor binding sequences, and for *per2*, these binding sequences are essential for *in vitro* amplitude response to DEX in cultured primary mesenchymal stem cells [25]. Whether the amplitude increase in *per1* in response to DEX is due to direct stimulation of the glucocorticoid receptor binding sequences in the *per1* gene, or is the secondary results of *per2* amplitude upregulation in our tissues is unclear. It is curious to see that in the liver *per1*, but not PER2 amplitude is upregulated in response to DEX treatment, indicating tissue specific regulation of *per1* and PER2 genes to DEX, and *per1* amplitude regulation independent of PER2. In *in vitro* cultures of primary mesenchymal stem cells, circadian rhythms in *per2* show larger circadian amplitude in transcription, as well as overall increased levels of transcription in response to DEX treatment [25]. Interestingly, a restraint induced stress response, also only upregulates *per1* mRNA in the heart and liver, and not *per2* [13].

Besides amplitude responses, we also looked at phase shift responses of PER2 reporting bioluminescence to medium and DEX treatment. We find that cultures made of atrial tissue show both a response to medium treatment and DEX treatment, but the treatment with DEX is always additive, and the *magnitude* of the phase response is similar to that reported for *in vivo* treatment of DEX in the liver [24]. To our knowledge, this is the first report of a time-dependent response to DEX in the atrium, or any other tissue than the liver.

As a positive control we also looked at the PRC to DEX treatment in the liver. We find that also the liver shows clear responses to medium and DEX treatment, but in the liver, the shapes of the respective phase response curves (PRCs) are much more different than they are in the atrium. Importantly, the direction of the phase responses (delays or advances) to DEX in the liver *in vitro* are as predicted from the *in vivo* DEX PRC [24], assuming that the peak phase of PER2 represents ∼CT18–19 [45]. In comparison to the atrial PRC to DEX treatment, the liver shows a significantly different type and size of response to DEX treatment. We also used the SCN as a negative control and as predicted this tissue exhibited limited responses to DEX treatment.

Interestingly, our data indicates the presence of a ‘dead zone’ in atrial phase responses to DEX, in which the stimulus does not result in a shift of the rhythm [24]; between 16–23 hours and 16–18 hours after the last peak in bioluminescence the observed DEX phase responses consist of minor variations around 0 hrs (with and without medium responses subtracted, respectively). This is in contrast to the liver, where the PRC for DEX treatment *in vivo* indicates the absence of such a large ‘dead zone’ [24]. However, our *in vitro* liver data does in fact show limited DEX induced phase shifts at 12.5–13.5 hours after the last peak in bioluminescence, suggesting the presence of an unresponsive component of the liver DEX PRC.

Medium treatment appears to have a smaller effect in the liver than in the atrium. In the atrium, where the medium treated PRC is relatively large and shows both advances and delays, the medium treated PRC may be have been due to the serum in the medium. However, we found no significant difference in response between 0, 1 and 5% serum in the fresh medium or treating with its own original medium. Also, the similar response to fresh medium and treatment with its own medium indicated that the response is not elicited by a factor that is present in the new medium. Only taking the culture out of the incubator does not result in a similar phase shift response, and opening the lid of the culture dish also does not result in a significant phase shift, suggesting that simply moving the culture, or gas exchange with the external environment is not the cause of the phase shift. Taken together, our results may suggest that some component of the medium treatment procedure, irrespective of the content, causes large shifts in the atrium. One explanation could be that the atrium has a high sensitivity for the mechanical stress it undergoes during the treatment. Such a hypothesis is intriguing, when taking into account the mechanical nature of cardiac tissue and the sensitivity of the atrial cardiomyocytes to stretch, and should be studied further. This hypothesis can also be adopted to explain the effects of timing of culturing (a mechanically stressful procedure), which also results in a larger range of phase shifts in the atrium, as compared to liver and SCN tissue. It may have some functional consequence in the intact animal with clock resetting of the heart, noting that day versus night differences in activity of the heart occur and are often associated with daily temporal exposure to exercise and stress. Furthermore, several components of the intracellular signaling cascade responsible for conveying the atrial myocardial stretch response for atrial natriuretic peptide secretion, are shared with those regulating the circadian clock, e.g. protein kinase C and mitogen-activated protein kinase (MAPK) [22], [32], [46]–[48].

Both the *per1* and *per2* genes contain glucocorticoid receptor binding sequences, making direct interaction between the glucocorticoid receptor and each gene promoter possible, and may be indicative that *per1* and *per2* are sites of action for DEX to alter the circadian clock [25]. Using liver specific knockout of the mouse circadian clock, Kornmann *et al* [38], [49] revealed that rhythmic expression of *per2* in the liver, is under the control of both the local clock and systemic cues. This makes *per2* an ideal candidate gene for an ‘entry point’ of systemic cues into the local liver clock. Our data indicate that indeed PER2 expression is altered in response to DEX in terms of phase in both the atrium and the liver, while *per1* does not show rhythmic expression in the atrium in the absence of external signals. In contrast, *per1* appears to be the more responsive of the two genes to DEX in both liver and atrium in terms of amplitude, while PER2 only shows an amplitude response in the atrium.

In the data presented here, we observe tissue dependent differences in *per1* and PER2 reported bioluminescence: 1) *per1* is not rhythmic in atria, while PER2 is rhythmic; 2) Amplitude responses in PER2 to DEX treatment are absent in liver and SCN, but show a clear time-dependent response in the atrium; 3) The PER2 phase response curve to DEX treatment in the atrium is significantly different from the liver. In addition there is a gene related observation, which is that we observe an amplitude response to DEX treatment in *per1*, but not PER2 in the liver, while in the atrium both *per1* and PER2 respond to DEX treatment with an amplitude increase. These tissue dependent observations are in line with the finding of tissue-dependent effects of *per2* and *per3* knockout mice [50]. Conversely, it has been shown that blood-borne cues can elicit circadian rhythm in liver and kidneys of animals bearing SCN lesions, but not in heart, spleen, or skeletal muscle of the same animals [51]. These data highlight the diversity and complexity of the local tissue circadian clocks.

As a last observation, we noticed a significant phase difference between the PER2 reporting bioluminescence peaks, where the right atrium is significantly delayed as compared the left atrium. In an earlier study by Davidson et al [16] cultures of the left ventricle appeared to have a delayed phase compared to those of the right atrium in *per1*-driven bioluminescence in the rat. This finding may indicate that the functional differences in the left and right atria and ventricles is reflected in phase angle differences between the clocks in these cardiac compartments.

The physiological relevance of our findings are several: firstly, the GR specific effect on atrial amplitude of canonical clock components would ensure a robust pacemaker, achieved either through individual cell changes in amplitude, or through increased coordinated rhythms in expression in a population of cells, or a combination of the two (see above). *In vivo*, normal GR signaling on a daily basis may be an important mechanism in the cardiac system for maintaining normal cardiomyocyte 24 hr rhythmicity. Secondly, whilst the phase resetting effect of GR signalling in the circadian clock has been demonstrated [22], little is known of this mechanism of entrainment beyond the hepatic system. Differences in the phase response curves to DEX between atria (heart) and liver may explain the phase differences in rhythms of certain canonical clock genes observed *in vivo* between these two tissues [11]. For example, inspection of the phase estimate data in this study [11] reveal *per1* and *per2* to peak at earlier circadian times in the heart compared to the liver, with a phase difference of approximately 3 hours. Thirdly, the implications of our data are implicit if we consider that DEX and other glucocorticoid drugs are used in the broad treatment of inflammatory and autoimmune conditions [52], [53]: the effects of such treatment paradigms could alter internal circadian coordination between different organ systems and between the heart and the day-night/activity-rest cycle, and therefore might impact cardiovascular health. Fourthly, the apparent sensitivity to mechanical change highlights the possibility that enhanced cardiac activity *in vivo*, such as during exercise or stress, might in itself result in a resetting of the circadian clock of the atria.

In summary, we find that the clock in atrial tissue shows a strong phase shifting effect in response to time of culturing. Together with the phase shift observed after medium treatment, this could lead to the hypothesis of a time-dependent sensitivity of the atrial clock to mechanical treatment, which is highly relevant for a tissue that shows a daily variation in mechanical (stretch and contractile) activity. Atrial tissue also shows phase shifting responses to DEX, which is markedly different from that of the liver. Moreover, in atrial tissue *per1^luc^* expression is not rhythmic, but can be induced by DEX, while PER2^LUC^ is rhythmic and shows a strong amplitude response at certain times within the circadian cycle. To our knowledge, this is the first report of a time dependent response to DEX in the atrium, or any tissue other than the liver. These data indicate that the clock in the atrium has a defined glucocorticoid sensitivity and circadian clock characteristics *in vitro*.

## Materials and Methods

### Ethics Statement

Animal experiments were approved by the University of Notre Dame Animal Care and Use Committee (protocol number 14–080) and performed in accordance with NIH Guidelines for the Care and Use of Laboratory Animals.

### Animals

Adult (age = 276±24 days, mean ± SEM) *per1*:*luciferase* (*per1^luc^*; N = 11; CD1 background; [54]) and PER2::LUCIFERASE (PER2^LUC^; N = 13; C57bl/6J background; [37]) mice were generated from in-house breeding at the University of Notre Dame. Food and water were available *ad libitum*. Animals were housed in 12 h light:12 h dark (LD) regimen under climate controlled conditions (19–21°C, 60–70% humidity). All mice were entrained to the LD regimen for at least 3 weeks, allowing all tissues to be stably entrained at the time of tissue culturing.

### Culturing and Treatment

Mice were sacrificed by cervical dislocation at 2–4 hours into the light period of the LD cycle (ZT 2–4; ZT 0 = lights on, ZT 12 = lights off). Brain, heart and liver tissue was quickly removed and placed in ice-cold Hanks Balanced Salt Solution (HBSS; Cellgro, Mediatech, Inc., VA, USA). Coronal sections of the brain (300 µm thickness) were cut on a manual vibroslice (NVSL; World Precision Instruments, Inc., FL, USA). Brain regions were identified under a dissecting microscope and the SCN was isolated as a square tissue (∼1.5 mm^2^), containing either a rostral or caudal aspect of the SCN. Liver tissue was also sectioned using the vibroslice at a thickness of 250 µm, and isolated in a square of approximately 1 mm^2^. Left and right atrial tissue was hand-cut with scalpel blades and reduced to small cubes (∼1 mm^3^), and in all experiments explants were taken from both right and left atria. We cultured only atrial, and not ventricular tissue, because we were unable to consistently generate ventricle explants that exhibited reliable rhythms in gene/protein expression.

Explants were cultured, using an established method, on a polypropylene mesh or permeable membrane, creating an interface between culture medium and humidified air and housed within sealed culture dishes [55]–[57]. SCN tissues were cultured on culture plate inserts (Millicell, Millipore, MA, USA), and atria and liver were placed on a mesh (Polypropelyne Spectra/Mesh [210 µm opening, 308 µm thickness, 34% open area], Spectrum laboratories,Inc., CA, USA). All tissue samples were placed in individual 35 mm translucent petri dishes with 1.2 ml of culture media composed of Dulbecco’s Modified Eagle’s Medium (Sigma-Aldrich), 19 mM D-glucose, 4.2 mM NaHCO3, 10 mM HEPES, 25 U/ml penicillin and streptomycin, 5% FBS and 100 mM luciferin (Luck-100, Gold Biotechnology, MO, USA), and sealed with vacuum grease and glass coverslips [57]. Tissues were placed in a light tight box at 36°C and bioluminescence was measured using a photomultiplier tube (LumiCycle, ActiMetrics, IL, USA) every 10 min.

As a reference, Table 1 and Figure 1 report on success rate of culturing and effects of time of culturing on the occurrence of the first peak in the rhythm. The analysis presented in this table and figure are based on all experiments conducted in our laboratory including the two mouse lines that are also used in the experiments reported here.

For treatment, samples were taken out of the incubator, but remained in the petri-dishes and were kept at a constant temperature of 37°C on a slide warmer during the media changing process. The culturing medium was aspirated off and kept in the dark at 37°C, to be returned after treatment. Tissues were given fresh culture medium, with or without 100 nM dexamethasone (DEX: Dexamethasone 21-phosphate disodium salt, Sigma-Aldrich, MO, USA) [24], without luciferin, for 30 min. During the incubation, samples were maintained in an incubator at 37°C. Subsequently, cultures were washed twice with 37°C culture media without luciferin each for 3–5 min and the original medium was returned. All tissues were subsequently placed back into the LumiCycle, for continued bioluminescence measurements.

### Experimental Design and Analysis

Our primary interest lay with atrial responses to DEX. As a reference SCN tissue was cultured as a tissue that does not respond to DEX, and the liver, as a tissue with some reported responses to DEX. Tissue cultures from both per1luc and PER2LUC mice were submitted to one of three experimental groups: no treatment, medium treated and DEX treated tissues. Tissues were treated at different times during the circadian cycle, and timing of the treatment was denoted as time since the last peak in bioluminescence driven by either per1 or PER2. The effect of treatment was assessed both for amplitude responses and phase changes of the bioluminescent signal. Amplitude changes in per1luc and PER2LUC reporting tissues were expressed as the ratio between the bioluminescence counts of the last pre-treatment peak to the first post-treatment peak. Phase changes in PER2LUC reporting tissues were calculated for medium and DEX treated cultures by comparing the first post-treatment peak in bioluminescence to that of the phase-matched no treatment control. Using this method, we controlled for possible variations in initial phase after the culturing procedure, e.g. due to culturing an explant derived from left or right atrium. In an additional experiment, we tested the magnitude of the phase shift of atrial tissues in response to an exchange in own culture medium and to exposure to fresh media of varying concentrations of 0, 1 and 5% fetal bovine serum versus only removal of the culture from the incubator. So as to mimic our basic medium/DEX protocol, in each case where medium was exchanged, cultures were washed twice with 37°C culture media without luciferin each for 5 min and the original medium was returned. To control for partial pressure changes in O2, CO2 and H2O in the air surrounding the surface of the tissue and culture medium, in a separate group lids were removed for 2 min to provide for air exchange, but otherwise the medium and explant in the culture dish were not disturbed.

### Peak/trough Determination

Recorded bioluminescence data were baseline subtracted (24 hour boxcar smoothing) and peaks and troughs were determined using an in-house algorithm detecting inflection points, described as follows. A low-pass filter was applied through boxcar smoothing on a 3 hour window size, thus removing small fluctuations outside the circadian range. A sliding set of two adjacent groups of 18 data points (representing 3 hours) was iteratively run through the dataset, moving forward one data point at a time. Forced linear regressions were calculated through each of the two groups of 18 data points and compared for the sign of the slope. An inflection point was called when the sign of the slope switched - with a ‘stability prerequisite’ that the slopes found in the preceding and following iteration did not show a sign change. A personal computer (PC) tool running this algorithm is available upon request.

### Northern Blot Analysis of Gene Expression in Mouse NIH3T3 Fibroblasts

Mouse NIH3T3 fibroblasts (ATCC) were grown in DMEM, 10% fetal calf serum and PSG at 37°c in 5% C02, and seeded as 2.5×105 cells/10 cm. Cells reached confluency after 4 days. After 7 days, cells were treated with 50% horse serum medium (in DMEM+PSG) for 2 hours, which was replaced with serum free DMEM+PSG for the duration of the experiment. The cells were taken at time = 0, 1 and every 4 hours for a 48 hour period (start of serum treatment was designated time = 0 h), rinsed with 1X PBS and total RNA harvested in 1 ml of Trizol (Gibco BRL) and purified following the Gibco BRL protocol. 20–40 µg of RNA was obtained per petri dish.

For Northern blot analysis, 10 µg of the extracted total RNA were electrophoresed on a 1% agarose gel containing 5% formaldehyde, and RNAs transferred to nitrocellulose membrane (Osmionics, Westborough, MA) by capillary transfer. For riboprobes the following templates were used: mPer1, 627–1681 (ORF); mPer2, 768–1643 (ORF); Riboprobes were synthesized using plasmids transcribed into antisense RNA riboprobes using Strip-EZ RNA kit (Ambion, Austin, TX) with T7 polymerase and NEN [alpha-32P] dUTP (6000 Ci/mmol) to a specific activity of 109 cpm/µg. Hybridization was carried out at 60–65°C, and blots washed according to standard techniques [23] and exposed to Kodak X-Omat Blue XB-1 film for 1–3 days. Densitometric determinations were made using Adobe PhotoShop after scanning films. Northern blot signals were normalized to ethidium bromide stained 28 S ribosomal band signals on the membrane. mRNA values were made relative the median mRNA value of the individual time course (1.0).

### Statistics

A comparison of the number of cultures that were deemed rhythmic or not rhythmic between *per1^luc^* and PER2^LUC^ was tested using a chi-square contingency table. Northern blot signals over the 48 h time courses were analyzed by one-factor ANOVA, followed by post-hoc Dunnett's t-tests. Amplitude and phase shift measures in response to treatment were tested using ANOVA’s with levels time, tissue and treatment where applicable. Post hoc analysis of significant main and interaction effects was conducted using the Holm-Sidak multiple pairwise comparisons test. Due to the non-equidistant nature of the data, phase shifts were averaged per 3 hours (atrium) and 6 hours (liver) for statistical analysis. A comparison between atrial and liver responses to medium and DEX treatment at different time of day was made by fitting a mixed linear model to the data (SAS, proc mixed). The timing of the amplitude responses to DEX in PER2^LUC^ bioluminescence was also tested by fitting a mixed linear model to the data (proc mixed) and phase differences between right and left atrium were tested using a Student’s t-test. All analyses were conducted using SAS version 9.2 (SAS Institute, Cary, NC, USA) or Sigmaplot version 11 (Systat Software, Inc., Chicago, IL, USA).

## Supporting Information

Figure S1 **Amplitude responses to medium and DEX treatment for cultures of** ***per1^luc^*** **mice.** Similar to PER2^LUC^ mice, *per1^luc^* SCN tissue did not show a change in amplitude in response to treatment. In contrast to PER2^LUC^ mice, *per1^luc^* liver tissue exhibited an increased amplitude after medium treatment, and a further increased amplitude after DEX treatment. Atrial tissue did not show a pre-treatment rhythm in bioluminescence, and therefore the increase in bioluminescence after treatment (see Figure 2) could not be quantified. N/A = not available, none = not treatment. **P<0.01.
(PDF)

Figure S2 **Phase shift responses to medium and DEX treatment of cultures of SCN tissue of** ***per1^luc^*** **and PER2^LUC^ mice.**
(PDF)

Figure S3 ***per1*** **and** ***per2*** **gene expression profiles in serum-stimulated mouse NIH3T3 fibroblasts.** (A) Representative Northern blots showing transcriptional profiles. Band sizes described at right in kilobases (kb). Mean ± SEM time course profiles for *per1* (B) and *per2* (C) gene expression. Data are represented as fraction of median expression (1.0) and values are plotted as mean ± SEM (N = 4–7 Northern blots). Both *per1* and *per2* were induced with 1 hour after serum treatment (one-way ANOVA, P<0.01 and P<0.001 for *per1* and *per2* respectively). After 8 hours *per1* levels were back at baseline and remained there, while in *per2* a circadian oscillation was induced, and mRNA levels peaked again 24 h and 48 h later (Dunnett's t-tests; P’s <0.05 at least).
(TIFF)
