# Supplementary figures and images for: Cardiac Atrial Circadian Rhythms in PERIOD2::LUCIFERASE and per1:luc Mice: Amplitude and Phase Responses to Glucocorticoid Signaling and Medium Treatment
Source: PLoS One. 2012 Oct 23;7(10):e47692. doi: 10.1371/journal.pone.0047692 (PMC3479129; doi:10.1371/journal.pone.0047692)

*per1<sup>luc</sup>* amplitude responses

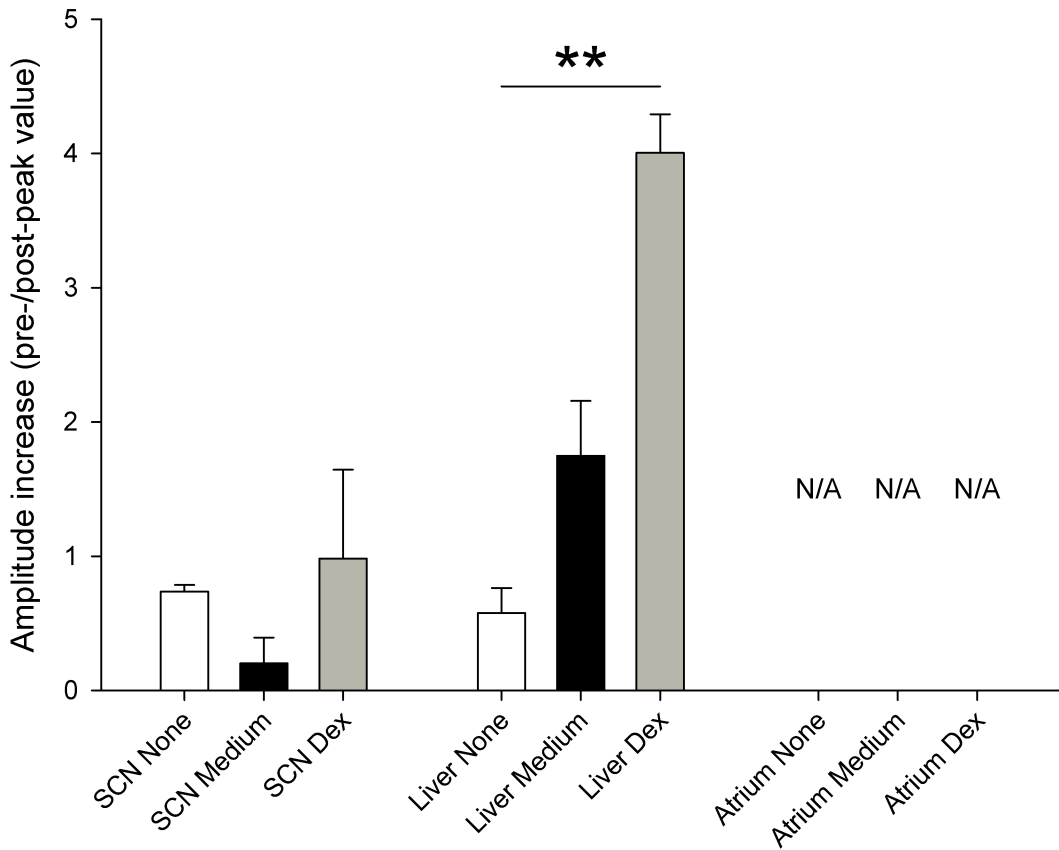

Supplement: Figure S1 — Amplitude responses to medium and DEX treatment for cultures of per1luc mice. Similar to PER2LUC mice, per1luc SCN tissue did not show a change in amplitude in response to treatment. In contrast to PER2LUC mice, per1luc liver tissue exhibited an increased amplitude after medium treatment, and a further increased amplitude after DEX treatment. Atrial tissue did not show a pre-treatment rhythm in bioluminescence, and therefore the increase in bioluminescence after treatment (see Figure 2) could not be quantified. N/A = not available, none = not treatment. **P<0.01. (PDF) [file pone.0047692.s001.pdf]

# Phase responses to medium and DEX treatment in SCN cultures

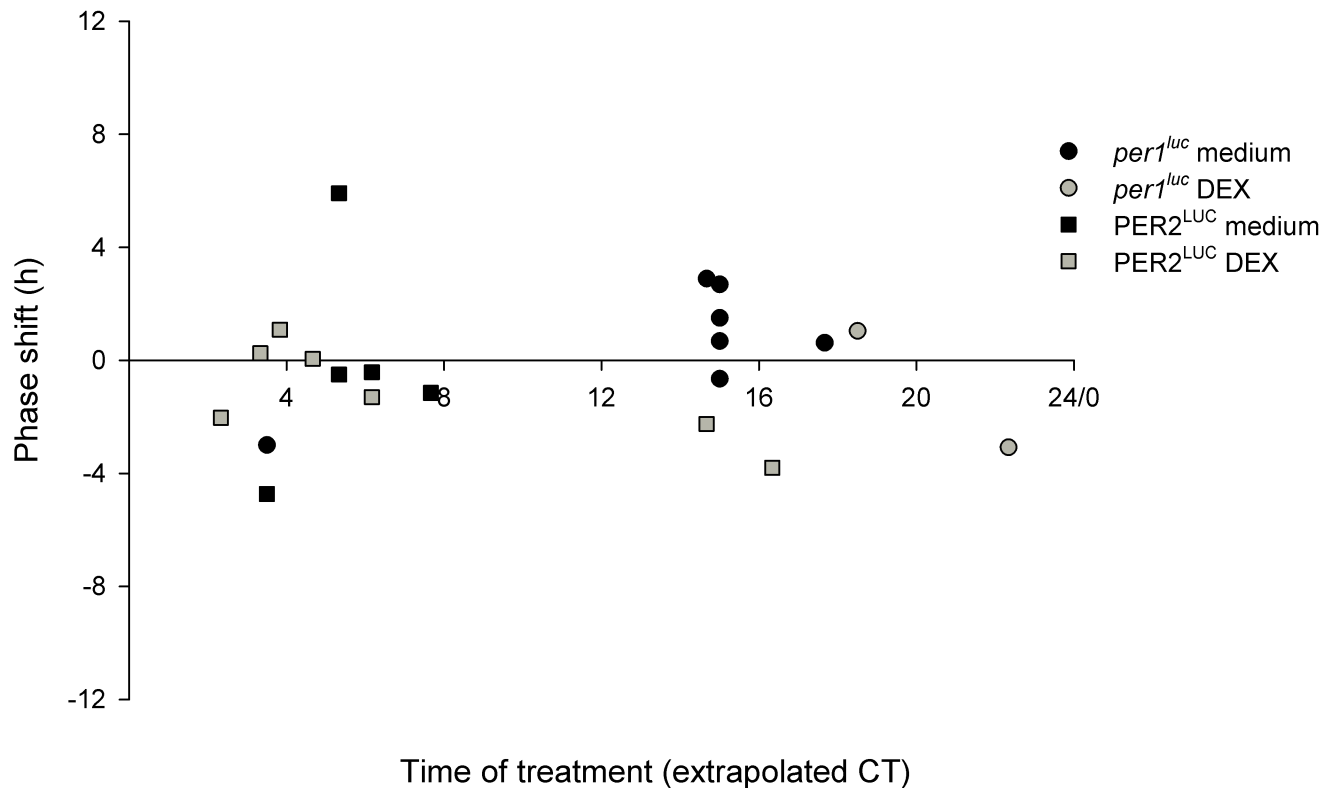

Supplement: Figure S2 — Phase shift responses to medium and DEX treatment of cultures of SCN tissue of per1luc and PER2LUC mice. (PDF) [file pone.0047692.s002.pdf]
